# A potent, broadly neutralizing human monoclonal antibody that efficiently protects hACE2-transgenic mice from infection with the Wuhan, BA.5, and XBB.1.5 SARS-CoV-2 variants

**DOI:** 10.3389/fimmu.2024.1442160

**Published:** 2024-07-19

**Authors:** Sergey V. Guselnikov, Konstantin O. Baranov, Sergey V. Kulemzin, Tatyana N. Belovezhets, Anton N. Chikaev, Svetlana V. Murasheva, Olga Y. Volkova, Ludmila V. Mechetina, Alexander M. Najakshin, Nikolai A. Chikaev, Pavel P. Solodkov, Maria V. Sergeeva, Alexander V. Smirnov, Irina A. Serova, Oleg L. Serov, Alexander G. Markhaev, Yulia V. Kononova, Alexander Y. Alekseev, Marina A. Gulyaeva, Daria M. Danilenko, Nariman R. Battulin, Alexander M. Shestopalov, Alexander V. Taranin

**Affiliations:** ^1^ Institute of Molecular and Cellular Biology, Siberian Branch of the Russian Academy of Sciences, Novosibirsk, Russia; ^2^ Department of Vaccinology, Smorodintsev Research Institute of Influenza, Saint Petersburg, Russia; ^3^ Department of Etiology and Epidemiology, Smorodintsev Research Institute of Influenza, Saint Petersburg, Russia; ^4^ Institute of Cytology and Genetics, Siberian Branch of the Russian Academy of Sciences, Novosibirsk, Russia; ^5^ Research Institute of Virology, Federal Research Center of Fundamental and Translational Medicine, Novosibirsk, Russia; ^6^ Department of Natural Sciences, Novosibirsk State University, Novosibirsk, Russia

**Keywords:** iC1 antibody, COVID-19, *in vivo* protection, Omicron, SARS-CoV-1

## Abstract

The COVID-19 pandemic has uncovered the high genetic variability of the SARS-CoV-2 virus and its ability to evade the immune responses that were induced by earlier viral variants. Only a few monoclonal antibodies that have been reported to date are capable of neutralizing a broad spectrum of SARS-CoV-2 variants. Here, we report the isolation of a new broadly neutralizing human monoclonal antibody, iC1. The antibody was identified through sorting the SARS-CoV-1 RBD-stained individual B cells that were isolated from the blood of a vaccinated donor following a breakthrough infection. *In vitro*, iC1 potently neutralizes pseudoviruses expressing a wide range of SARS-CoV-2 Spike variants, including those of the XBB sublineage. In an hACE2-transgenic mouse model, iC1 provided effective protection against the Wuhan strain of the virus as well as the BA.5 and XBB.1.5 variants. Therefore, iC1 can be considered as a potential component of the broadly neutralizing antibody cocktails resisting the SARS-CoV-2 mutation escape.

## 1 Introduction

Despite the declared end of the COVID-19 global health emergency, there remains a need for effective therapy, particularly for at-risk groups, such as immunocompromised or immunodeficient individuals. Human monoclonal antibodies neutralizing SARS-CoV-2 have become one of the most important tools of counteracting coronavirus infection in this population. Since November 2020, drugs such as the cocktail of casirivimab and imdevimab (1), the combination of bamlanivimab (2) and etesevimab (3), sotrovimab (4), bebtelovimab (5), regdanvimab (6) as well as the cocktail of tixagevimab and cilgavimab (7) (Evusheld) have been authorized for emergency use (8). However, at present, the authorization has been revoked due to the emergence and spread of new viral variants of the Omicron lineage capable of evading the immune response elicited by the original Wuhan-1 variant of SARS-CoV-2 (9–13). Moreover, out of thousands of monoclonal antibodies against SARS-CoV-2 characterized to date, only a few have been shown to neutralize the broad spectrum of the viral variants, including the BQ, XBB, or JN lineages (14–17).

**Figure 1:**
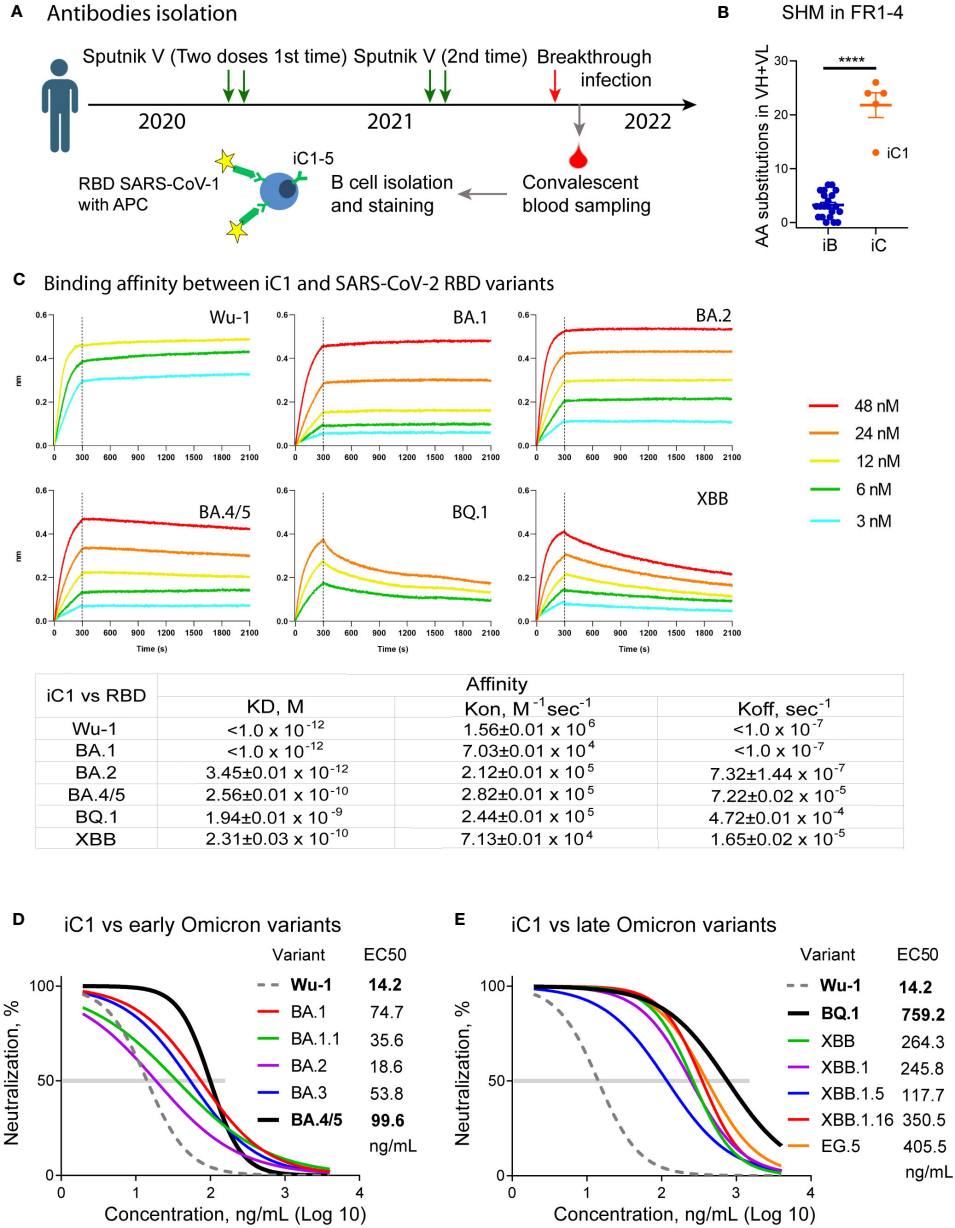
Isolation and primary characterization of the iC series of SARS-CoV-2 binding antibodies. Nucleotide sequences encoding iC1-5 antibodies were obtained from single-sorted B cells of a previously vaccinated COVID-19 convalescent individual using single cell PCR **(A)**. The iC antibodies underwent more intensive somatic hypermutation (SHM) compared to the iB antibodies (18) (**** – p<0.0001) **(B)**. BLI analysis of the binding affinity between iC1 and SARS-CoV-2 RBD variants shows picomolar to nanomolar values **(C)**. *In vitro* pseudovirus neutralization by the iC1 antibody demonstrates its broad neutralizing activity **(D, E)**.

We previously reported the isolation of a panel of monoclonal antibodies capable of neutralizing the Wuhan-1 variant of SARS-CoV-2 with ultrahigh potency (18). Of these, one was shown to neutralize both the early viral variants and the Omicron variant BA.1–BA.4/5 (19). In the present study, we set out to isolate SARS-CoV-2-neutralizing antibodies that have a broader range using an individual with hybrid antiviral immunity as the source of B cells (**Figure 1A**). The choice of such a donor was dictated by the data showing that hybrid immunity induced by multiple exposure to vaccine antigens and live SARS-CoV-2 typically displays broader neutralization (20–22).

## 2 Materials and methods

### 2.1 Single B cell sorting, cloning, and antibody production

Peripheral blood mononuclear cell isolation and single B cell sorting were described previously (18) except for the use of the Spike protein of SARS-CoV-1 (23). Single-cell cDNA synthesis, reverse transcription PCR, and cloning of antibody variable sequences into pAbVec expression vectors encoding constant regions of human γ1, κ, or λ chains were performed according to (24, 25). Antibodies were produced and purified as described (18).

### 2.2 Biolayer interferometry

Measurements of Kd, Kon, Koff, and epitope binning for purified antibodies were performed as described previously (18) using OctetRed96 (Fortebio, Sartorius, Germany) with SARS-CoV-2-S-RBD-His6 immobilized on NTA biosensors (Cat #18-5101). The following human reference antibodies with known epitopes were used via BLI for epitope binning (competition assay): bamlanivimab (2), cilgavimab (7), sotrovimab (26), bebtelovimab (5), imdevimab (1), tixagevimab (7), casirivimab (1), CV30 (27), S2X259 (28), S2H97 (29), S2K146 (30), and SA55 (31). The reference antibodies were produced in-house based on the published VH and VL sequences (32).

### 2.3 SARS-CoV-2 Spike-pseudotyped lentivirus neutralization assay

SARS-CoV-2 Spike-pseudotyped lentiviral particles were produced as described previously (18). HEK293T cells were transfected with a 4:6:3 molar mixture of plasmids psPAX2, pCDHNLuc, and a pCAGGS-SpikeΔ19 plasmid encoding either a Wuhan-1 (wild-type) or a mutant Spike variant without the 19 C-terminal residues. The constructs encoding wild-type and mutant variants of the Spike protein were obtained either via gene synthesis (Genewiz, USA; NovoPro Bioscience, China; Evrogen, Russia) or PCR mutagenesis with sets of mutagenic primers (33). A Spike-pseudotyped lentivirus neutralization assay was performed as described (19). Briefly, ACE2-HEK293T cells were transduced with a mixture of the antibody and the Spike-pseudotyped lentiviral particles; 48 hours later, the cells were washed, lysed, and luminescence intensity upon the addition of the substrate (1.25 µg of freshly prepared h-coelenterazine (Nanolight Technologies, Germany) in 50 µL of PBS per well, 3 s) was measured. The half-maximal inhibitory concentration (IC50) was determined by non-linear regression as the concentration of antibody that neutralized 50% of the pseudotyped lentivirus.

### 2.4 SARS-CoV-2 isolates

Three SARS-CoV-2 isolates were used for *in vivo* experiments in hACE2-transgenic mice. Preparation of the SARS-CoV-2/human/RUS/Nsk-FRCFTM-1/2020 isolate (EPI_ISL_481284) which is characterized by a single D614G substitution in the Spike protein (lineage B.1) has been described previously (18). SARS-CoV-2 isolates hCoV-19/Russia/SPE-RII-MH71262/2022 (BA.5.2 lineage, GISAIDEPI_ISL_14596294) and hCoV-19/Russia/SPE-RII-9714/2023 (XBB.1.5 lineage, GISAID EPI_ISL_16902719) were cultured from nasopharyngeal swabs in VeroE6/TMPRSS2 cells (JCRB, #JCRB1819). The cells were inoculated for two hours with swab material diluted 1/10 in DMEM (Biolot, Russia) supplemented with 2% heat-inactivated fetal bovine serum (Biolot, Russia), 1% antibiotic-antimycotic (Gibco, Thermo Fisher Scientific, USA), and then incubated for 4-5 days until 70-100% cytopathic effect. Following a single round of viral passaging, a working viral stock was obtained. Virus titers were measured using the standard TCID50 method.

### 2.5 *In vivo* protection assay

C57BL/6-Tg (CAG-ACE2)5Nrba/Icg mice were generated through a pronuclear injection of a genetic construct containing the cDNA of the human *ACE2* gene under the control of a strong ubiquitous constitutive chimeric CAG promoter (34). The transcription of the hACE2 cassette was observed in all organs of these mice, and hACE2 protein was detected on the cell surface. The infection of mouse embryonic fibroblasts with SARS-CoV-2 infection by the Wuhan-1 variant resulted in pronounced cytopathic effects. In these transgenic mice, signs of viral infection were observed in the lungs following intranasal infection. No signs of infection in the brain were observed when analyzing the histological sections (34). The mice were obtained from the SPF animal facility of the Institute of Cytology and Genetics SB RAS (Novosibirsk, Russia). The animals were weighed. The values ranged from 15.1 to 26.1 g. In the prophylactic scheme, mice were intraperitoneally (i/p) administered with 10 mg/kg iC1 (group 1, n=5; group 3, n=5; group 5, n=6) or total human IgG as a negative control (group 2, n=4; group 4, n=3; group 6, n=5) 24 h before infection (–1 dpi). On the next day, (0 dpi) SARS-CoV-2 was given intranasally (50 μL/nostril) at a total dose of 3.0-3.3 lgTCID50/mouse of the B.1 (groups 1 and 2), BA.5 (groups 3 and 4), and XBB.1.5 (groups 5 and 6) virus variants. Untreated animals (group 7, n=6) were used as an additional baseline control. In the therapeutic regimen, 14 mice were infected as above with XBB.1.5 and administered iC1 (group 8, n=8) or a placebo (group 9, n=6) 6 h post-infection (10 mg/kg, i/p). The animals were monitored for any signs of distress and weighed daily. The animals were euthanized on 9 dpi.The lungs from the mice infected with the Wuhan and XBB.1.5 variants were extracted for pathology analysis; blood samples were collected to measure the levels of human IgGs in the serum. Assessment of the levels of injected human antibodies in the sera of infected mice was performed as described (18).

### 2.6 Statistical analysis

Statistical analysis was performed in GraphPad Prism 8: The significance of differences between groups was tested with Mann Whitney test (**Figure 1B**); Nonlinear regression was used for the neutralization curves transformation and IC50 determination (**Figures 1D, E**).

## 3 Results and discussion

A peripheral blood sample was collected from a donor on day 14 following a laboratory-confirmed breakthrough infection with SARS-CoV-2 (**Figure 1A**). The infection occurred at the time of the BA.1/BA.2 Omicron surge in February 2022 (35). However, the exact variant of the virus was not determined. To sort the individual B-cells, we used fluorescein-labeled receptor-binding domain (RBD) from the related sarbecovirus SARS-CoV-1, which was the causative agent of the 2002 coronavirus outbreak. The donor had no history of previous exposure to SARS-CoV-1. While it is now clear that the binding of antibodies to SARS-CoV-1 does not guarantee a high neutralization breadth (17), we anticipated that such an approach would help identify antibodies targeting conserved RBD epitopes. As a result, five monoclonal antibodies (iC1-5) were isolated. The new iC-series antibodies exhibited a high level of somatic hypermutation (8 to 19 amino acid substitutions in the framework regions of a VH and 2 to 11 substitutions in the framework regions of a VL), thereby indicating their prolonged maturation compared to the monoclonal antibodies isolated at the start of pandemia (18) (**Figure 1B**).

To assess the breadth of neutralizing activity of the obtained antibodies, we used lentiviral particles pseudotyped with the Spike protein of the Wuhan-1 variant as well as with the Spike of the various variants of Omicron lineage, including BA.1, BA.1.1, BA.2, BA.3, BA.4/5, BQ.1, XBB, XBB.1, XBB.1.5, XBB.1.16, EG.5, and SARS-CoV-1. Despite the fact that donor B cells were sorted based on their binding to SARS-CoV-1 RBD, none of the isolated monoclonal antibodies appreciably neutralized SARS-CoV-1 Spike-pseudotyped lentiviral particles. Two antibodies, iC1 and iC4, showed neutralizing activity against several variants of SARS-CoV-2 pseudoviruses. Of these, iC4 neutralized the BA.1, BA.2, and BA.3 variants with IC50 of 311, 109, and 975 ng/mL (not shown). In contrast, iC1 potently neutralized all of the SARS-CoV-2 variants tested (**Figures 1D, E**) and was pursued for further studies with the highest activity against Wu-1 (IC50 = 14 ng/mL) and the lowest activity against BQ.1 (759 ng/mL). We also assessed the dissociation constants of the iC1 complexes with RBD of the Wu-1, BA.1, BA.2, BA.4/5, BQ.1, and XBB variants. The antibody bound these RBD with KD ranging from 1.94E-09 (BQ.1) to ~1.0E-12 (Wu-1 and BA.1) (**Figure 1C**).

**Figure 2:**
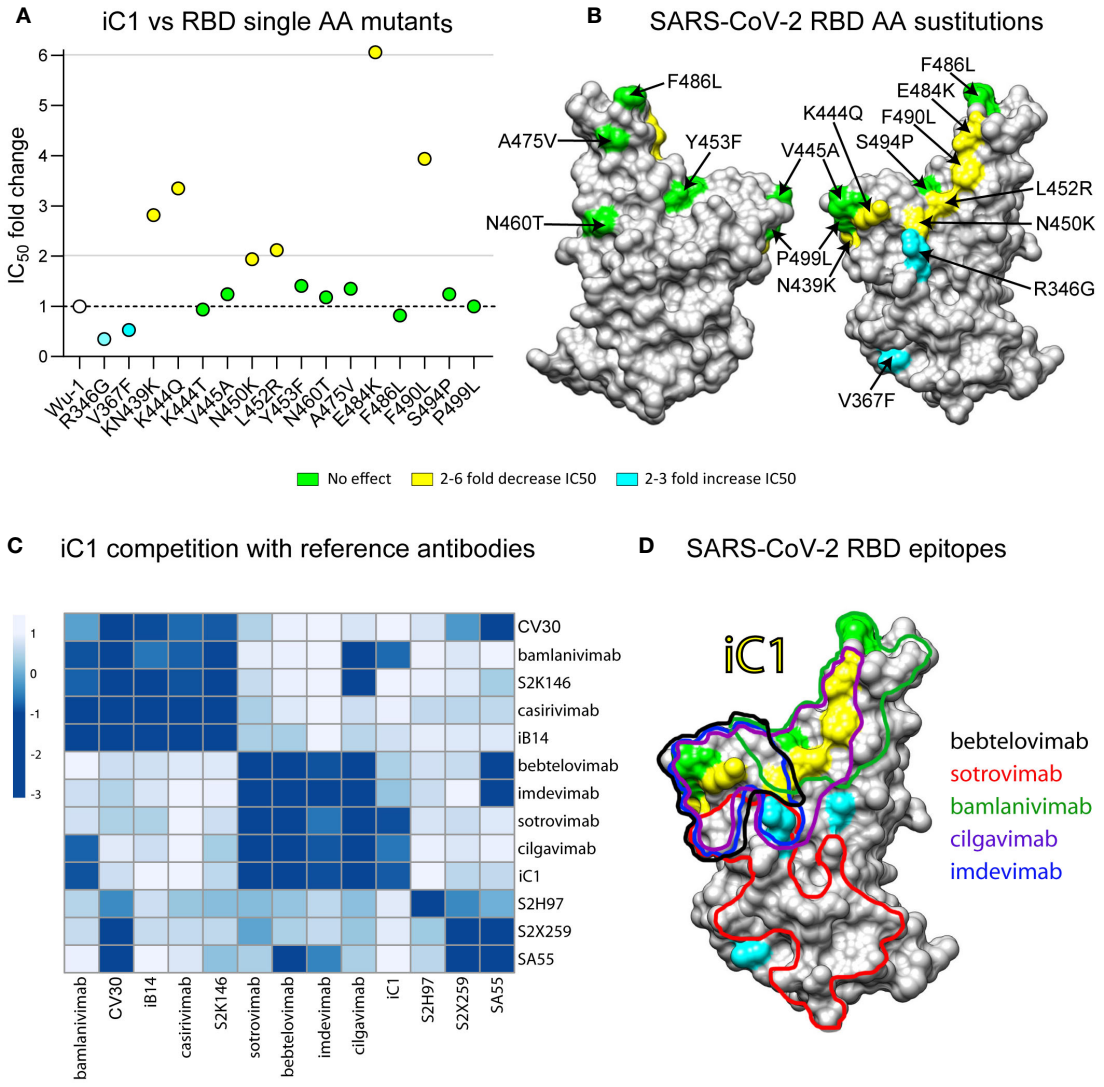
Epitope mapping of iC1 binding to the RBD of SARS-CoV-2. Neutralizing activity on a panel of 15 pseudoviruses with the Spike protein (Wuhan-1) variant carrying single amino acid substitutions in the RBD region **(A)**. A 3D model of the RBD (SARS-CoV-2) with highlighted substitutions causing effect on iC1 neutralizing activity (yellow/blue) **(B)**. BLI analysis of iC1 competition with 12 reference antibodies with known epitope structures. Negative values (dark blue color) mean that there is a competition, positive values (white/light blue color) means that there is no competition **(C)**. An overlay of the epitopes for the reference antibodies with single amino acid residues influencing the iC1 neutralizing activity **(D)**.

For the epitope mapping of the antibody iC1, we assessed its neutralizing activity on a panel of 15 pseudoviruses with the Spike protein of the ancestral Wuhan-1 variant carrying single amino acid substitutions in the RBD region. None of the substitutions that were tested has resulted in the complete loss of iC1 neutralizing activity. Notably, the substitutions N439K, K444Q, L452R, F490L, and E484K caused a twofold to sixfold decrease in iC1 neutralizing activity. Conversely, the substitutions R346G and V367F increased antibody activity twofold and threefold, respectively (**Figures 2A, B**). Amino acid residues, whose substitutions affected iC1 activity, clustered on the outer face of the RBD and are likely a part of the RBD-iC1 complex interface. This assumption is further supported by the results of the BLI analysis of iC1 competition with 12 reference antibodies whose epitope structures are known. The incubation of iC1 with RBD completely blocked the binding of antibodies targeting the outer face (36) region of RBD, including bamlanivimab, cilgavimab, sotrovimab, bebtelovimab, and imdevimab (**Figure 2C**) (1, 2, 5, 7, 26). In reciprocal setups, bamlanivimab, cilgavimab, and to a lesser extent sotrovimab blocked the interaction of iC1 with RBD. The binding of bebtelovimab and imdevimab as the first antibody had a negligible effect on the ability of iC1 to interact with RBD. At the same time, iC1 did not compete for binding to RBD with the reference antibodies tixagevimab, casirivimab, CV30, S2X259, S2H97, S2K146, and SA55, which are known to predominantly interact with various regions of the inner face of RBD (**Figure 2C**) (1, 7, 27–31). Taken together, these results suggest that the epitope of iC1 maps to the upper part of the outer face of RBD, and it has an overlap with the epitopes of bamlanivimab and cilgavimab as well as partially with that of the sotrovimab (**Figure 2D**). However, the broader range of neutralization of iC1 clearly shows that its epitope is different from those of bamlanivimab, cilgavimab and sotrovimab. The absence of competition with the antibodies binding to the inner face region of RBD indicates that iC1 can potentially be used in a cocktail with such antibodies to counteract SARS-CoV-2 mutation escape.

**Figure 3:**
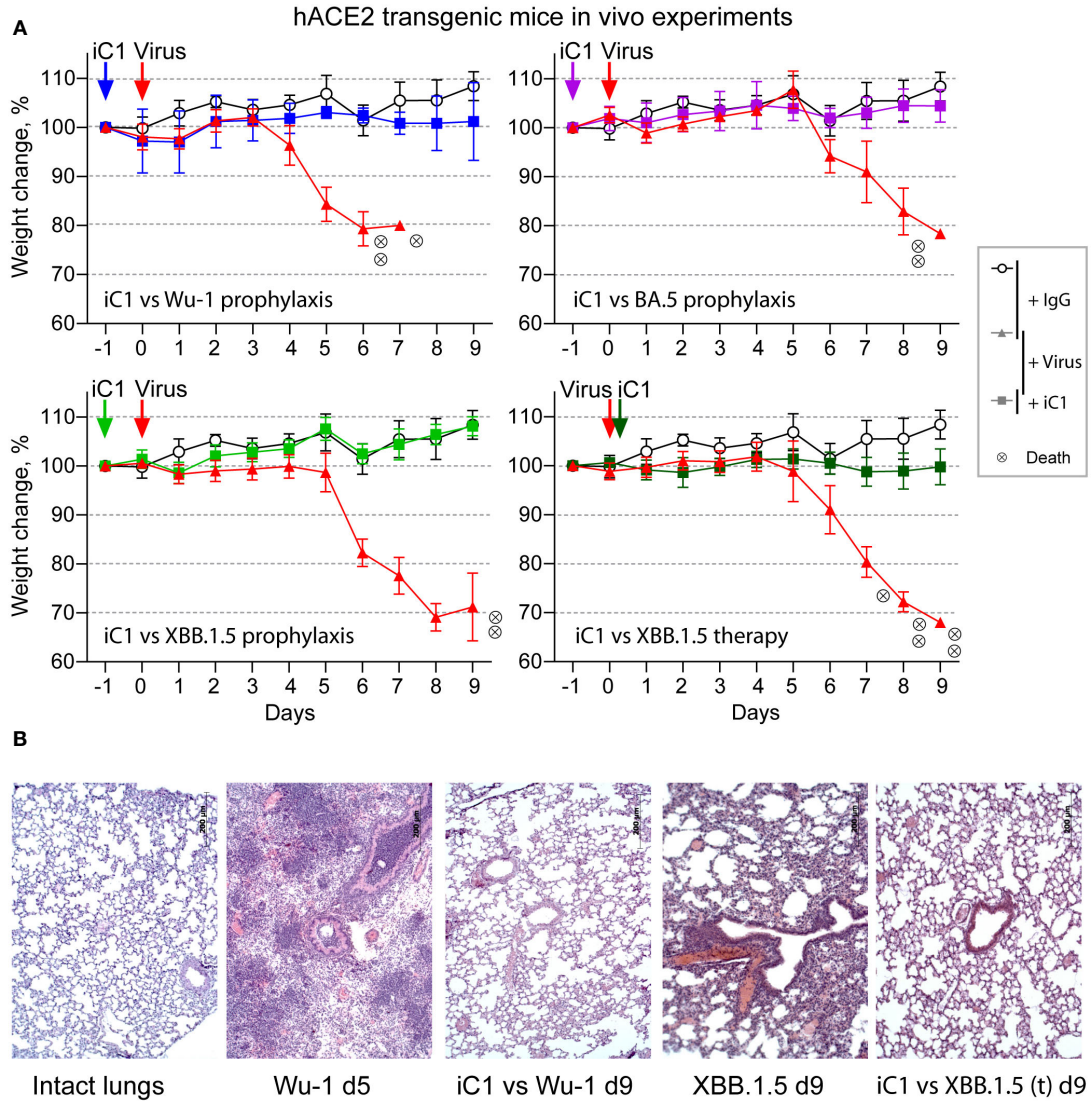
*In vivo* activity of iC1 (hACE2 transgenic mouse model). Prophylactic and therapeutic properties of iC1 antibody against Wu-1, BA.5, and XBB.1.5 authentic SARS-CoV-2 viruses **(A)**. The open circles represent placebo-treated mice (human IgG only), the red triangles represent the negative control mice (IgG+virus), and the color squares represent the experimental mice (iC1+virus). Each circle with an “x” indicates death of one mouse. Histological analysis of the lungs of mice infected with the Wu-1 and XBB.1.5 variants **(B)**. The mouse treated with Wu-1 and a placebo was sacrificed on day 5 due to its moribund state, and all other mice were sacrificed at the end of the experiment on day 9.

iC1 only showed moderate potency when tested against the XBB and BQ virus variants, with IC50 values ranging from 118 to 759 ng/mL (**Figure 1E**). However, it is well recognized in the field that the *in vivo* protective activity of the antibodies against SARS-CoV-2 may differ from that demonstrated in *in vitro* tests (37–39). To evaluate the therapeutic and prophylactic properties of iC1, we studied its effect on infection in hACE2-transgenic mice. In addition to the Wuhan-1 variant virus, two variants of the Omicron lineage, BA.5 and XBB.1.5, were tested *in vivo*. The infection of placebo-administered animals with the Wuhan-1 variant in the prophylactic scheme led to rapid weight loss. One mouse from this group was sacrificed on the 5th day in order to examine the lung pathology. The rest of the group died by day 8. In the case of BA.5, two mice died, and one lost 22% of its weight by day 9. When infected with XBB.1.5, two mice died, and three mice had a weight loss of 21-32% by day 9. In the therapeutic experiment, three XBB.1.5-infected and placebo-treated mice died, and three lost 31-33% of their weight by day 9 (**Figure 3A**). The milder course of infection in mice infected with BA.5 and XBB.1.5 is consistent with the data on the reduced pathogenicity of Omicron sublineages compared to the earlier viral variants (40, 41).

The prophylactic administration of iC1 at a dose of 10 mg per kg body weight 24 h before infection with either viral variant completely prevented not only the death of the mice but also their weight loss. In the therapeutic setting, the administration of iC1 6 h after infection with the XBB.1.5 variant also prevented the weight loss and death of the experimental mice (**Figure 3A**). Histological analysis of the lungs taken from the control mice infected with the Wuhan and XBB.1.5 variants showed the development of severe hemorrhages. Prophylactic administration of iC1 before infection with the Wuhan-1 variant largely prevented these complications (**Figure 3B**). Similar effect was observed in XBB.1.5-infected mice after therapeutic treatment with iC1.

After the completion of the described experiments, a new SARS-CoV-2 variant, BA.2.86, has emerged. This variant, in turn, has initiated the widespread dissemination of the JN.1 variant and several of its subvariants. Compared to its predecessor BA.2, the JN.1 variant exhibits 33 mutations in the Spike protein, including 13 substitutions and one deletion in the RBD (42). Notably, the RBD mutations in JN.1 include substitutions at positions that, according to our findings, may be part of the epitope recognized by iC1, specifically R346T, G446S, N450D, and L452W. Our recent analysis has demonstrated that iC1 does not neutralize a pseudovirus carrying the JN.1 Spike (data not shown). Regardless of whether the loss of such activity is a result of the cumulative effect of Spike substitutions or specific individual mutation(s), the data indicate that the JN.1 variant is likely resistant to neutralization by iC1.

## 4 Conclusion

In summary, in the present study, we describe a novel SARS-CoV-2-specific human monoclonal antibody, iC1. This antibody exhibits excellent protective properties *in vivo* against not only the Wuhan-1 variant of SARS-CoV-2 but also against the BA.5 and XBB.1.5 variants. Although iC1 does not appear to neutralize the currently dominant sublineage JN.1, it belongs to a group of rare SARS-CoV-2-specific antibodies with the highest neutralization breadth. Considering the significant likelihood of the future emergence of SARS-CoV-2 variants that are antigenically distinct from the JN.1 lineage (43), it is conceivable that iC1 antibody may still find application as a potential component of broadly neutralizing antibody cocktails for countering the mutation escape of SARS-CoV-2.

## Data availability statement

The original contributions presented in the study are included in the article/supplementary material. Further inquiries can be directed to the corresponding author.

## Ethics statement

The animal study was approved by Committee on Biomedical Ethics of the Federal Research Center for Fundamental and Translational Medicine (Novosibirsk, Russia). The study was conducted in accordance with the local legislation and institutional requirements.

## Author contributions

SG: Data curation, Formal analysis, Investigation, Project administration, Visualization, Writing – original draft, Writing – review & editing. KB: Formal analysis, Investigation, Methodology, Visualization, Writing – review & editing. SK: Conceptualization, Data curation, Formal analysis, Investigation, Project administration, Writing – original draft, Writing – review & editing, Visualization. TB: Investigation, Writing – review & editing. AC: Investigation, Writing – review & editing. SM: Writing – review & editing, Investigation. OV: Investigation, Writing – review & editing. LM: Investigation, Resources, Writing – review & editing. AN: Investigation, Writing – review & editing. NC: Investigation, Writing – review & editing. PS: Investigation, Writing – review & editing. MS: Writing – review & editing, Resources. AS: Methodology, Writing – review & editing. IS: Methodology, Writing – review & editing. OS: Methodology, Writing – review & editing. AM: Investigation, Writing – review & editing. YK: Investigation, Writing – review & editing. AA: Investigation, Writing – review & editing. MG: Investigation, Writing – review & editing. DD: Resources, Supervision, Writing – review & editing. NB: Methodology, Supervision, Writing – review & editing. AS: Investigation, Supervision, Writing – review & editing. AT: Funding acquisition, Supervision, Writing – original draft, Writing – review & editing.
